# Clinical and imaging features of mixed Alzheimer and vascular pathologies

**DOI:** 10.1186/s13195-015-0104-7

**Published:** 2015-02-27

**Authors:** Helena C Chui, Liliana Ramirez-Gomez

**Affiliations:** Department of Neurology, University of Southern California, 1570 Alcazar Street, Suite 215, Los Angeles, CA 90033 USA

## Abstract

The co-occurrence of both Alzheimer disease (AD) pathology and vascular brain injury (VBI) is very common, especially amongst the oldest of old. In neuropathologic studies, the prevalence of AD, VBI, and mixed AD/VBI lesions ranks ahead of Lewy bodies and hippocampal sclerosis. In the modern era of structural magnetic resonance imaging (MRI) and amyloid positron emission tomography (PET) imaging, this review examines 1) the prevalence of mixed AD and VBI pathology, 2) the significance of these pathologies for cognitive impairment (AD and vascular cognitive impairment (VCI)), and 3) the diagnosis and treatment of mixed AD/VCI. Although epidemiologic studies report that vascular risk factors for arteriosclerosis increase the risk of incident AD, both autopsy and amyloid PET studies indicate that AD and VBI contribute additively, but independently, to the risk of dementia. The literature confirms the malignancy of AD and highlights the adverse effects of microinfarcts on cognitive function. For the clinical diagnosis of mixed AD/VCI, the presence of AD can be recognized by neuropsychological profile, structural imaging, cerebrospinal fluid biomarkers, and glucose PET and amyloid PET imaging. The diagnosis of VBI, however, still hinges predominantly on the structural MRI findings. Severe amnesia and atrophy of the hippocampus are characteristic of early AD, whereas the cognitive profile for VCI is highly variable and dependent on size and location of VBI. The cognitive profile of mixed AD/VBI is dominated by AD. With the notable exception of microinfarcts (which elude *in vivo* detection), infarcts, hemorrhages, and white matter hyperintensities on structural MRI currently represent the best markers for the presence VBI. Better markers that reflect the health and reactivity of intracerebral blood vessels are needed. For prevention and treatment, the type of underlying cerebrovascular disease (for example, arteriosclerosis or cerebral amyloid angiopathy) should be considered. It is likely that reduction of vascular risk factors for arteriosclerosis can significantly reduce vascular contributions to mixed dementia.

## Introduction

Traditionally, Alzheimer disease (AD) and vascular dementia are recognized as the two most prevalent forms of dementia in late life. A combination of AD and vascular pathologies (so-called mixed dementia) is usually registered as a close third, moving up to first or second in rank in community-based studies of the oldest of old. Conceptualization and diagnosis of these entities has been evolving from clinical-pathological phenotypes, which could not be resolved until autopsy, to new research diagnostic criteria that incorporate molecular biomarkers (for example, amyloid-beta (Aβ) and phosphorylated tau), and *in vivo* structural, functional, and perfusion imaging. Neuropsychological features (for example, pattern and severity of impairment across principle cognitive domains) remain relevant to diagnosis and clinical care. Innovations are underway in computerized neuropsychological assessment (for example, computerized assessment with continuous measures, such as the National Institutes of Health tool box) and rethinking cognitive impairment in terms of functional neural networks. In this review, we consider mainly how recent advances in biomarkers and imaging have altered our conceptualization and diagnosis of mixed AD and vascular pathologies.

Medically speaking, the approach to mixed AD and vascular pathologies should be tied to prevention and treatment. During the past 30 years, emphasis has moved from dementia to mild cognitive impairment to preclinical disease, in the hopes that prevention and treatment can be instituted earlier in the disease course. AD is currently conceptualized as a combined amyloidopathy and tau-related neurodegeneration. Mainstream strategies to prevent and treat AD target these misfolded proteins.

**Figure 1:**
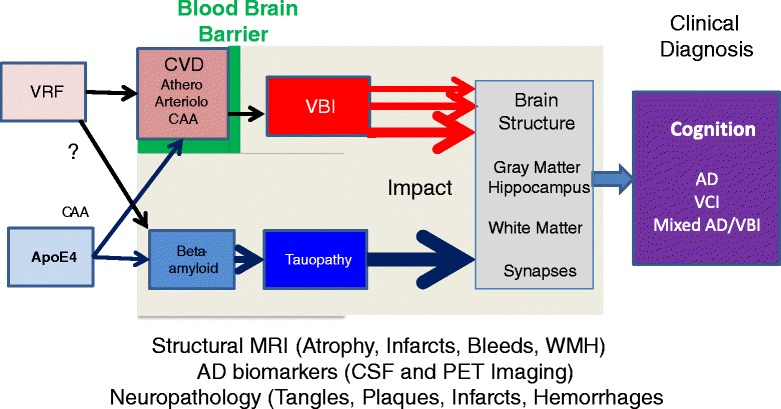
**Mixed Alzheimer disease/vascular brain injury.** Additive parallel or interactive pathways? AD, Alzheimer disease; ApoE, apolipoprotein E; CAA, cerebral amyloid angiopathy; CSF, cerebrospinal fluid; CVD, cerebrovascular disease; MRI, magnetic resonance imaging; PET, positron emission tomography; VBI, vascular brain injury; VCI, vascular cognitive impairment; VRF, vascular risk factor; WMH, white matter hyperintensity.

The model for vascular cognitive impairment (VCI) adopted here posits that vascular risk factors (VRFs) lead to cerebrovascular disease (CVD), which leads to vascular brain injury (VBI), which leads to VCI (Figure 1). It is paramount to maintain focus on the type of CVD (the cause), as well as on the resulting VBI and VCI. For example, the prevention and treatment of cerebral amyloid angiopathy (CAA), which is intrinsically associated with AD, is likely to differ fundamentally from the prevention and treatment of atherosclerosis. It may pay off to pay some attention to terminology, rather than to lump all vascular dementias into the category of VCI.

In this review of clinical and imaging features of mixed AD/VCI, we have chosen nuanced terminology to distinguish levels of vascular factors: mixed AD/VRFs, mixed AD/CVD, mixed AD/VBI, and mixed AD/VCI. We use the term VCI when clinical criteria are used to define groups, VBI when study groups are defined by infarcts/hemorrhages noted by imaging/pathology, and CVD to refer to specific disorders of blood vessels (for example, atherosclerosis or amyloid angiopathy). Finally, we use the term VRFs to refer to traditional risk factors for atherosclerosis (for example, hypertension, diabetes mellitus, and hyperlipidemia) and not for amyloid angiopathy (for example, apolipoprotein E (ApoE) ε4 is mentioned distinctly and not included under the label VRF in this review).

The diagnosis of VCI is reflected in recent clinical criteria [1], which draw heavily on evidence of infarcts, white matter hyperintensities (WMHs) and microbleeds (MBs) using structural magnetic resonance imaging (MRI). Multiple autopsy studies have shown that microinfarcts (one type of VBI) are major risk factors for VCI; however, microinfarcts fall below the resolution of 1.5 and 3 T MRI. This is one of several gaps in *in vivo* detection that we must close going forward.

For the diagnosis of AD in this review, we adopt research-level criteria for high likelihood of preclinical, mild cognitive impairment and dementia due to AD based on biomarker evidence [2-4]. This requires evidence of 1) amyloid deposition by autopsy, low cerebrospinal fluid (CSF) Aβ, or positive amyloid positron emission tomography (PET) and 2) neurodegeneration by autopsy, high CSF ptau, and AD pattern of atrophy on structural imaging. While amyloid and tau biomarkers are not required in clinical practice for the diagnosis of AD, this review is intentionally more selective.

In attempting to define clinical and imaging features of entities that are defined by clinical and imaging phenotype, we must be aware of possible circular reasoning. For example, in seeking to characterize neuropsychological features, we have selected study groups based on mixed AD/VBI, not mixed AD/VCI (unless the diagnosis of VCI was reached without knowledge of the neuropsychological profile).

Diagnostic classifications such as AD, VCI and mixed AD/VCI represent an oversimplified dichotomous framework, necessary for clinical practice. With the expanding repertoire of neuroimaging modalities, it makes sense in the long term to adopt a multi-factorial model that plugs in continuous measures of AD and VBI as independent variables and looks to continuous measures of various cognitive domains as the outcome. Indeed, many of the studies reviewed below used multi-factorial models where imaging features are correlated with cognitive performance.

We are now at the infancy of testing whether these cognitive outcomes are mediated by injury to corresponding cognitive networks (using functional MRI and diffuse tensor imaging) and how these systems change over time. We look forward to visualizing how AD pathology and VBI impact neural networks and how treatment might protect, sustain, and repair them.

## Prevalence of mixed AD/VCI or mixed AD/VBI in prospective longitudinal autopsy studies

Over four decades ago, the high prevalence of AD and vascular pathology in older patients (mean age = 76.4 years) was brought to light by Tomlinson, Blessed, and Roth [5,6]. Previously, AD had been considered a relatively rare cause of early onset dementia, while arteriosclerosis was widely held to be the most common cause of late onset dementia. Among 50 cases with dementia living in a mental hospital, widespread plaques and tangles (that is, AD changes) were the predominant pathological lesions in 50%, while cerebral softening (that is, territorial infarcts) due to atherosclerosis accounted for 12 to 17%, and mixed AD and VBI pathologies were found in 8 to 18%. At this juncture, the pendulum of clinical diagnosis and investigation swung dramatically from arteriosclerotic dementia to AD.

In 1997, three decades later, the Nun Study heightened interest in mixed dementia, by showing additive effects of AD and VBI on risk of dementia [7]. The Nun Study introduced a generation of prospective, longitudinal, clinic-to-autopsy cohort studies which have systematically addressed clinical-pathological correlations in dementia (Tables 1 and 2). We review some of these studies from the United States, United Kingdom, and Japan. The prevalence of mixed AD/VBI among cases with dementia ranges from 14 to 44%.

**Table 1:** **Community-based, autopsy studies are required to estimate the prevalence and incidence of mixed Alzheimer disease/vascular brain injury**

**Autopsy study**	**N**	**Mean age (years)**	**Diagnosis of AD**	**Diagnosis of VBI or VCI**	**Prevalence of mixed**	**Interaction between VBI and AD on risk of dementia**	**Neuropsychology**
Nun Study [7]	102	87 (76-100)	Khachaturian plaque criteria	Number of infarcts > or <1.5 cm	39% (24/61) of dementia cases were mixed	Number of tangles and number of lacunes exert independent additive effect on MMSE and likelihood of dementia	MMSE
							CERAD
ROS [8,9]	550	87	Mean number of neurofibrillary tangles, neuritic plaques, and diffuse plaques in five lobes	Number of macroscopic and microscopic infarcts	28% of dementia cases were mixed	AD and VBI pathology have additive effect on odds of dementia	
MAP [8,9]	425	87	Mean number of neurofibrillary tangles, neuritic plaques, and diffuse plaques in five lobes	Number of macroscopic and microscopic infarcts	44% of dementia cases were mixed		
BLSA [10]	179	87.6 ± 7.1	CERAD	Macroscopic infarcts	Hemispheral infarcts alone or with AD account for 35% of dementia cases	In subjects with intermediate AD pathology scores, a single macroscopic hemispheral infarct was sufficient to cause dementia	Blessed Memory Information Concentration Test
			Braak and Braak stage	Microscopic infarcts			
BLSA [11]	200			Atherosclerosis (0-3) of coronary, aorta, and intracranial vessels	45% have remote infarct	68% of cases have atherosclerosis, which increased the odds of dementia independent of AD pathology or cerebral infarcts	
	175 complete autopsies, including heart and aorta						
MRC CFAS [12]	N = 456	87 (SD = 7): range 66 to 100 (63% ≥85)	CERAD scale (0-3)	Regional infarcts (>1 cm)		Association between AD pathology and cognitive status goes down with age	MMSE
	243 dementia		Diffuse plaques, neuritic plaques, tangles, atrophy				AGECAT
	183 without dementia			Small vessel disease: lacunes, microinfarcts, white matter change			
						Association between atrophy and age continues to go up	
	30 unknown						
MRC CFAS [13]				Self-reported vascular risk factors		Vascular risk factors were not associated with an increased burden of AD pathology at death in old age	
				26% of non dementia cases had CVA; 43% of dementia cases had CVA			
CC75 + C [14]	224	91	CERAD		22% of 113 dementia cases		
Hisayama [15]	N = 469		CERAD	NINDS-AIREN	4.7% of dementia cases were mixed		
	275 incident dementia cases		NIA-Reagan				
	(164 autopsies)						
HAAS [16]	N = 443	86 ± 5.2	Mean number of neurofibrillary tangles and neuritic plaques over 20 fields in 4 lobes	High correlations noted between MBIs and lacunar infarcts (Spearman r = 0.45, *P* < 0.0001)	14.2% of dementia cases were mixed	No correlation between AD and microvascular lesions	CASI
		(72-90+)					
HAAS [17]	N = 436			Number of infarcts > or <1.0 cm, microinfarcts		MBI found in 72% of demented and 61% of non-demented	
	144 with dementia						
	292 without dementia					MBI and AD exert independent additive effects on cognition	

AD, Alzheimer disease; AGECAT, Automated Geriatric Examination for Computer Assisted Taxonomy; BLSA, Baltimore Longitudinal Aging Study; CASI, Cognitive Abilities Screening Instrument; CC75 + C, Cambridge City Over-75 s Cohort; CERAD, Consortium to Establish a Registry for Alzheimer Disease; CFAS, Cognitive Function and Ageing Study; CVA, cerebrovascular accident; HAAS, Honolulu Asia Aging Study; MAP, Rush Memory and Aging Project; MBI, microscopic brain infarct; MMSE, Mini-Mental State Exam; MRC, Medical Research Council; NIA, National Institute on Aging; NINDS-AIREN, National Institute of Neurological Disorders and Stroke-Association Internationale pour la Recherche et l’Enseignement en Neurosciences; ROS, Rush Religious Order Study; SD, standard deviation; VBI, vascular brain injury; VCI, vascular cognitive impairment.

**Table 2:** **Subcortical Ischemic Vascular Dementia (SIVD) Neuropathology study: imaging and clinical correlations**

**Autopsy study**	**N/pathological diagnosis**	**Mean age (years)**	**Diagnosis of AD**	**Diagnosis of VBI**	**Prevalence of mixed**	**Neuropsychology**	**Interaction between VBI and AD on risk of dementia**
Subcortical Ischemic Vascular Dementia (SIVD) study [18]	N = 61	78 (at time of neuropsych)	CERAD	CVD-PS		Linear composite measures	AD patient scores were lower than EXEC by nearly a standard deviation on average. VBI patients were rather equally impaired on EXEC,MEM and NVMEM
	AD = 23		Braak and Braak ≥ IV	Infarct score ≥20			
	VBI = 11					GLOB	
	Mixed = 9					MEM	
	NSP = 19					EXEC	
SIVD study [19]	N = 79	82.8 ± 7.0	NINDS-ADRDA criteria	ADDTC criteria	Clin Dx = 28%		Positive likelihood ratios: AD = 6.4 > VCI = 3.7 > mixed = 2.3
	AD = 34	At time of death					
	VBI = 15		CERAD	CVD-PS	Path Dx = 11%	Cognitive status (CN, CIND, Dem)	AD pathology and hippocampal sclerosis have relatively greater effect on cognition than VBI
	Mix = 9		Braak and Braak ≥ IV	Infarct score ≥20			
	NSP = 21						
SIVD study [20]	N = 93	84	Lacunes associated with WMH				
	Normal = 12		AD, atherosclerosis, and VBI contribute independently to cortical gray matter atrophy				
	AD = 46		AD and hippocampal sclerosis are associated with hippocampal atrophy				
	VBI = 14						
	Mixed = 9						
	NSP = 12						
SIVD study [21]	N = 163	84	Cerebral atherosclerosis was positively associated with microinfarcts (OR, 2.3; 95% CI, 1.2-4.4) and cystic infarcts (OR, 2.0; 95% CI, 1.0-4.2) but not AD pathology				
	Normal = 23						
	AD = 81		Cerebral amyloid angiopathy was inversely associated with lacunar infarcts (OR, 0.6; 95% CI, 0.41-1.1), but positively associated with Braak and Braak stage (OR, 1.5; 95% CI, 1.1-2.1) and Consortium to Establish a Registry for Alzheimer Disease plaque score (OR, 1.5; 95% CI, 1.1-2.2)				
	VBI = 21						
	Mixed = 15						
	NSP = 23						
SIVD study (Zheng *et al*., manuscript submitted for publication)	N = 116	84	Pathological measures of AD, atherosclerosis, and VBI contribute independently to MRI cortical gray matter atrophy and cognitive impairment. Path analyses show that the adverse effects of atherosclerosis on cognition are largely mediated through subcortical infarcts to brain atrophy, while the effects of AD on cognition work equally through cortical atrophy as well as through a yet unmeasured direct pathway				
	Normal = 12						
	AD = 53						
	VBI = 18						
	Mixed = 9						
	NSP = 24						

AD, Alzheimer disease; ADDTC, Alzheimer’s Disease Diagnostic and Treatment Centers; CERAD, Consortium to Establish a Registry for Alzheimer Disease; CI, confidence interval; CIND, Cognitive impairment not meeting criteria for dementia; Clin Dx, clinical diagnosis; CN, cognitively normal; CVD-PS, cerebrovascular disease-parenchymal score; Dem, dementia; EXEC, executive score; GLOB, global cognition score; MEM, memory score; NINDS-ADRDA, National Institute of Neurological Disorders and Stroke-Association Internationale pour la Recherche et l’Enseignement en Neurosciences; NSP, non-significant pathology; NVMEM, non-verbal memory; OR, odds ratio; Path Dx, pathological diagnosis; VBI, vascular brain injury; WMH, white matter hyperintensity.

The Rush Religious Orders Study (ROS) and the Rush Memory and Aging Project (MAP) constitute parallel, but independent, longitudinal clinical-pathological studies. Cases of clinically diagnosed probable AD often revealed a combination of AD plus other pathologies, especially VBI [8,22]. In the combined ROS and MAP autopsy samples (n = 804), mixed AD/VBI lesions were found in 16% of cases between ages 65 and 89 years, and grew to 28% after age 90 years [23]. Among cases with dementia, the prevalence of mixed AD/VCI was higher in the community-based MAP cohort (44%) than in the religiously defined ROS cohort (28%) [8]. The high prevalence of mixed AD/VBI and AD/VCI, especially in the oldest of old, underscores the importance of reducing VRFs as a public health priority.

In the Baltimore Longitudinal Aging Study (BLSA; n = 200), AD pathology alone accounted for 50% of the dementia seen in this cohort; hemispheral infarcts alone (VBI) or in conjunction with AD pathology (AD/VBI) accounted for 35%. In subjects with intermediate AD pathology scores, a single macroscopic hemispheral infarct was sufficient to cause dementia [10]. Atherosclerosis, brain infarcts, and AD pathology all contributed independently to risk of dementia [11]. Atherosclerosis scores were not correlated with AD pathology.

In the population-based Medical Research Council Cognitive Function and Ageing Study (CFAS; n = 456), the association between AD pathology (neuritic plaques and tangles) and cognitive status went down with age, while the association between atrophy and age continued to go up [12]. Self-reported VRFs were associated with infarcts, but not AD pathology [13]. Specifically, hypertension and heart attack were associated with microinfarcts in both dementia and non-dementia cases. In the Cambridge City over-75 s cohort, 22% of 118 dementia cases were classified as mixed AD/VBI [14].

In the population-based Hisayama Study, among 275 cases of incident dementia followed to autopsy, 45% were classified as AD, 30% as VCI, 5% as mixed AD/VCI and 4% as dementia with Lewy bodies [15]. The incidences of AD, combined dementia and other types of dementia rose with increasing age, particularly after the age of 85 years; this tendency was not observed for VCI or dementia with Lewy bodies. In an earlier autopsy study (n = 135), diabetes mellitus and insulin resistance were associated with neuritic plaques, but not neurofibrillary tangles [24].

In the Honolulu Asia Aging Study (HAAS) of older Japanese American men (n = 443 autopsies), microvascular lesions were the predominant lesion in 33% of dementia cases, AD the predominant lesion in 18.6% and mixed lesions (most often AD and VBI) in 14.2% [16]. The frequency of AD pathology and brain atrophy increased steadily after age 72 years (12 to 35% and 25 to 63%, respectively), whereas the frequency of microvascular lesions remained fairly constant at around 30% across the older age spectrum. Lewy bodies and hippocampal sclerosis jumped up in frequency to 15% and 10%, respectively, after age 80 years. The ratio of VBI to AD has been relatively higher in the HAAS cohort compared with other cohorts, raising the question of possible ethnic differences in gene-environment-brain interactions.

Based on the above community and cohort studies of prevalence, we conclude that mixed AD/VBI ranks in the top three most prevalent pathologies (with AD and VCI), well ahead of dementia with Lewy bodies and hippocampal sclerosis. Several studies have noted that the prevalence of AD pathology and atrophy continue to grow with advancing age, while the prevalence of infarcts remains more constant across the younger, middle and oldest of old. The prevalence of VBI compared with AD is higher in studies of Japanese (Hisayama and HAAS) compared with studies of predominantly Caucasians in the United States and United Kingdom (ROS, MAP, BLSA, CFAS, Cambridge City over-75 s cohort). However, among infarcts of various sizes, regardless of ethnicity, microinfarcts show the strongest correlation with cognitive impairment (HAAS, ROS, CFAS, BLSA). Consistently, autopsy studies have demonstrated that VBI and AD exert independent and additive effects on the risk of dementia.

## Weighting the relative effects of VBI, AD, and other pathological lesions on cognition

Beyond knowing the relative prevalence of pathological lesions, it is essential to weight their importance for cognitive impairment. The two variables (prevalence and clinical relevance) are not necessarily correlated. In the combined Rush ROS and MAP (n = 856) [25], subjects with normal cognition at enrollment who were followed longitudinally (mean 7.5 years) had a high prevalence of neuropathological findings at autopsy (mean age 88 years): 99% evinced plaques or tangles; 36% had at least one gross infarct; 28% had at least one microinfarct; and 11% had neocortical Lewy bodies.

The relative weighting of these pathological lesions on longitudinal cognitive decline was also studied: 22% of the rate of decline was explained by global AD pathology, 6% by amyloid plaques, 34% by tangles, 2% by macroscopic infarcts, and 8% by neocortical Lewy bodies. When analyzed in conjunction, all pathological indices explained 41% of the total variation in cognitive performance. In these two studies combined, the rank order significance of pathologic lesions for cognitive decline was: tangles > Lewy bodies > amyloid plaques > macroscopic infarcts.

The prevalence and impact of microinfarcts was examined in the ROS (n = 425 autopsies) [26]. Microinfarcts were present in 36.5% of persons with dementia and 25.3% in persons without dementia. The presence of microinfarcts, especially in multiple cortical locations, increased the odds of dementia (odds ratio, 1.77; 95% confidence interval, 1.07 to 2.92) and lowered average global cognition (estimate, -0.287; standard error (SE), 0.113; *P* = 0.012). Microinfarcts were associated (in order of effect size) with lower perceptual speed (estimate, -0.400; SE, 0.117; *P* < 0.001), semantic memory (estimate, -0.391; SE, 0.130; *P* = 0.003), and episodic memory (estimate, -0.279; SE, 0.138; *P* = 0.044). These associations were not modified by the presence of macroscopic infarcts or AD pathology, suggesting that the effects of microinfarcts were independent. Of relevance, 58 of 129 (45%) people with microinfarcts did not have macroscopic infarcts, reminding us that we cannot rely on MRI to exclude VBI.

In the HAAS, 65% of autopsied cases harbored microscopic brain infarcts (MBIs), which contributed significantly and independently to brain atrophy and cognitive impairment, particularly before dementia was clinically evident [17]. Scalars were developed to represent the severity of five different pathologic lesions (AD, MBI, hippocampal sclerosis, Lewy bodies, and atrophy). Spearman rank correlations (r) between these scalars and the last Cognitive Abilities Screening Instrument score were all significant, as follows (in rank order): atrophy, r = -0.453; AD lesions, r = -0.299; hippocampal sclerosis, r = -0.200; MBI, r = -0.195; and Lewy bodies, r = -0.158 [27]. The five types of pathology explained 40% of the variance in the last Cognitive Abilities Screening Instrument score [17].

In the Subcortical Ischemic Vascular Dementia study, a cohort was recruited from university-affiliated memory clinics, enriched for people who had lacunes and WMHs on MRI (Table 2). The correlation between level of cognitive impairment was far stronger with AD pathology and hippocampal sclerosis than VBI [19]. The profile of cognitive impairment for neuropathologically defined mixed AD/subcortical vascular dementia (SVD) resembled that seen in AD cases (memory scores were lower than executive scores by nearly one standard deviation) rather than SVD (where all cognitive domains were impaired more or less equally) [18]. These finding suggest that, in general, when SVD is combined with AD, the effects of AD on severity and profile of cognitive impairment overwhelm those contributed by SVD.

Analyses of these longitudinal, clinical, neuropathological, and imaging studies have moved our understanding from prevalence to clinical relevance of various pathological hallmarks. These data confirm the malignancy of AD pathology and highlight the importance of microinfarcts as one form of VBI. They demonstrate the usefulness of multivariate, continuous approaches to understanding brain-behavior relationships. At the same time, they indicate the limitations of current neuroimaging and neuropathological measures to model and predict cognitive decline.

## Pathological correlates of structural MRI in mixed AD/VBI

The advent of structural imaging computerized tomography (CT) scans in the 1970s and MRI scans in the 1980s) revolutionized our ability to visualize regional atrophy and large and small infarcts as well as WMHs and MBs [28]. A few longitudinal studies have attempted to validate structural MRI measures (for example, atrophy, WMHs) in reference to neuropathologic lesions and cognitive impairment. Pointedly, however, MRI scans at 1.5 and 3 T are not able to visualize microinfarcts, a form of VBI with demonstrated relevance to cognitive impairment. Recently, structural MRI at 7 T has revealed larger microinfarcts (for example, 0.7 mm diameter), whereas average microinfarcts (0.3 mm diameter) still fall below the detection threshold [29].

A variety of pathological changes in the brain parenchymal and vasculature have been ascribed to WMHs in late life (reviewed in [30]). Smooth, periventricular rims and punctuate lesions appear to have little clinical relevance. Irregular and confluent WMHs are correlated with a host of parenchymal changes (for example, variable loss of myelin and axons, and microglial and inflammatory changes) as well as a spectrum of vascular pathologies (for example, arteriolosclerosis, amyloid angiopathy, dilated perivascular spaces) [30]. Deep WMH lesions are thought to result from chronic hypoperfusion and hypoxia in terminal vascular beds fed by long, penetrating arterioles [31] and/or from breakdown of the blood-brain barrier and activation of matrix metalloproteinases [32,33]. In the Oregon Brain Aging Study, arteriolosclerosis (as opposed to atherosclerosis) was found to be the strongest correlate of WMHs [34].

In patients with AD, loss of fractional anisotropy, and increased mean and radial diffusivity are seen in white matter association tracts, especially the corpus callosum and cingulate and uncinate fasciculi [35,36]. These changes may be seen in the absence of WMHs, and most likely reflect secondary (Wallerian) degeneration from loss of cortical neurons/axons, but to some degree there is also a component of primary white matter degeneration [37]. The co-occurrence of WMHs in AD is commonly associated with the ApoE ε4 genotype, CAA, or arteriolosclerosis [30,34]. Thus, for the most part, WMHs in late life can be viewed as a marker for white matter change related to vasculopathy.

In the Subcortical Ischemic Vascular Dementia study, volumes of cortical gray matter, WMHs, hippocampus, and lacunes were obtained from structural MRI and correlated with pathology (Table 2). WMHs were straightforwardly associated with pathologic measures of vascular white matter injury; discrete lesions >3 mm and brighter than CSF correlated well with lacunar infarcts. On the other hand, neuropathology correlations for hippocampal volume and cortical gray matter were more complex. Hippocampal sclerosis and AD pathology explained 33% of the variance in hippocampal volume. A combination of AD pathology, arteriosclerosis, and subcortical VBI explained 25% of the variance in cortical gray matter [20].

To improve the clinical diagnosis of mixed AD/VBI, these findings suggest the requirement for a multi-variable and multi-modality algorithm. Structural MRI measures alone have limited sensitivity and specificity. WMHs, infarcts, and hemorrhages on MRI may be taken as suitable markers for VBI (minus microinfarcts).

Hippocampal volume is a suitable marker for AD (but could also mean hippocampal sclerosis). MRI atrophy, at least as a global measure, cannot be relied upon to differentiate a neurodegenerative versus vascular etiology. Several measures of amyloid and tau pathology (for example, amyloid and tau PET or CSF biomarkers) have been validated and would improve diagnostic specificity for AD.

### Differentiation of mixed AD/VBI in the era of amyloid PET imaging

Prior to the arrival of amyloid PET imaging [38] and the validation of CSF biomarkers [39], autopsy was essential to disclose the neuropathological hallmarks of AD (namely plaques and tangles). Hence our decision to begin a review of mixed AD/VCI with neuropathologically defined AD (Tables 1 and 2). During the past few years, however, the detection of early AD changes beginning in preclinical stages has been improved by amyloid PET ligands (for example, Pittsburgh Imaging Compound B (PiB), florbetapir, flutemetamole, and so on) [40,41].

A team of investigators at Samsung Medical Center, Seoul, Korea, separated patients with severe WMHs (evidence of SVD) into two groups based on positive or negative retention of amyloid on PiB PET scans (Table 3). Mixed AD/SVD (31% of 45 patients) performed worse on delayed recall, had fewer lacunar infarcts and had greater hippocampal atrophy than pure SVD [42]. The group went on to test the ability of MRI shape analysis to discriminate mixed AD/SVD from pure SVD [43]. Among 68 patients with SVD defined by severe WMHs, 23 (33.8%) patients were defined as mixed AD/SVD based on positive PiB binding. With use of hippocampal shape analysis alone, mixed SVD could be distinguished from pure SVD with 95.7% sensitivity and 68.9% specificity. With use of amygdalar shape, the discrimination accuracy was 87.0% sensitivity and 68.9% specificity. The two groups could also be distinguished based on the shape of the hippocampus and amygdala.

**Table 3:** **Studies of mixed Alzheimer disease/vascular brain injury with Alzheimer disease defined by amyloid PET imaging**

**Study**	**N**	**Age (years)**	**AD = PiB+**	**VBI**	**Mixed AD/VBI**	**Mixed AD/VBI findings**
Samsung [42]	N = 45 SVD severe WMH	74.2 ± 7.6		Pure SVD	Mixed AD/SVD	Mixed AD/SVD performed worse on delayed recall, had fewer lacunar infarcts and had greater hippocampal atrophy than pure SVD.
				N = 31 (69%) PiB-	N = 14 (31%) PiB+	
Samsung [43]	N = 68 SVD			Pure SVD	Mixed AD/SVD	Differentiation of mixed AD/SVD from pure SVD:
				N = 45 (66.2%)	N = 23 (33.8%)	Hippocampal shape analysis: 95.7% sensitivity; 68.9% specificity
				PiB-	PiB+	
						Amygdalar shape analysis: 87.0% sensitivity; 68.9%
Aging Brain Study [44]	N = 54	79	PiB+	VBI: WMHs and infarcts		No relationship between VBI (infarcts or WMHs) and PiB
						VBI is associated with impairment in EXEC
			N = 33 PiB-	N = 27 VBI-		No relationship between PiB and cognition
			N = 21 PiB+	N = 27 VBI+		
Aging Brain Study [45]	N = 61	79		VBI: Infarcts		Infarction, particularly in cortical and subcortical gray matter, was associated with lower cognitive performance in all domains (*P* < 0.05 for all comparisons)
			N = 32 PiB-	N = 27 infarct-		
			N = 29 PiB+	N = 34 infarct+		
Aging Brain Study [46]	N = 43	78.9 (6.7)	FCRP accounted for 16% of the variance in PiB index (*P* < 0.008) and the positive association remained significant controlling for age and sex, and apolipoprotein E genotype			
Aging Brain Study [47]	N = 74	79	Higher LDL-C and lower HDL-C levels were both associated with a higher PiB index, independent of apolipoprotein E genotype			
	33 NCI					
	38 MCI					
	3 CDR1					
Aging Brain Study [48]	N = 67	79	A relationship between Aβ and memory was mediated by cortical thickness.			
	35 NCI		The relationship between Aβ and cortical thickness was eliminated after controlling for FCRP, except in PiB+ subjects (n = 22), where Aβ remained associated with thinner cortex in precuneus and occipital lobe			
	31 MCI					
	1 CDR1		Vascular risk and Aβ both contribute to cortical thickness			

Aβ, amyloid-beta; AD, Alzheimer disease; CDR, Clinical Dementia Rating; EXEC, executive score; FCRP, Framingham Coronary Risk Profile; HDL-C, high density lipoprotein cholesterol; LDL-C, low density lipoprotein-cholesterol; MCI, mild cognitive impairment; NCI, no cognitive impairment; PET, positron emission tomography; PiB, Pittsburgh Imaging Compound B; SVD, subcortical vascular dementia; VBI, vascular brain injury; WMH, white matter hyperintensity.

In the Aging Brain Study [44,45], a longitudinal cohort enriched for vascular disease, no associations were found between measures of VBI on MRI and amyloid retention on PiB PET (Table 3). Participants with infarcts showed lower executive functioning (*P* = 0.001). Subcortical infarcts were inversely associated with declines in executive and memory function, whereas cortical infarcts were mainly associated with decline in executive function. Global PiB retention was associated with diminished non-verbal memory. Within this spectrum of normal aging to mild dementia, VBI and Aβ aggregation appeared to be independent processes with VBI having a greater impact on cognition than PiB retention. These studies illustrate how amyloid PET imaging will greatly improve our ability to recognize mixed AD/VCI cases of dementia.

### Differentiation of mixed AD/VBI with CSF biomarkers

CSF biomarkers for Aβ42 and tau also provide molecular evidence for AD, but the findings are more difficult to interpret. In the Amsterdam Dementia Cohort, the presence of both MBs and WMHs was associated with lower CSF levels of Aβ42, while lacunar infarcts were associated with higher Aβ42 and lower tau (Table 4) [49]. The investigators concluded that the data supported a direct relationship between SVD and AD pathology. However, if type of SVD is considered (that is, atherosclerosis versus CAA), the direct relationship between MBs and WMHs and AD biomarkers might reflect underlying CAA and AD, driven in common by ApoE ε4 genotype.

**Table 4:** **Studies of mixed Alzheimer disease/vascular brain injury with Alzheimer disease defined by cerebrospinal fluid Aβ and phosphorylated tau**

**Study**	**N**	**AD =**	**VCI/VBI**	**VBI and AD correlations**
ADNI [50]	N = 819	NINDS-ADRDA	VRFs	VRFs were not associated with AD biomarkers
	229 NCI		WMH	Increased time-varying WMHs were associated with faster decline in executive function and lower FDG uptake in NCI
	397 CI			
	193 AD			
Amsterdam Dementia Cohort [49]	N = 914	NINDS-ADRDA	VCI by NINDS-AIREN criteria	The presence of both MBs and WMHs was associated with lower CSF levels of Aβ42, indicating a direct relationship between SVD and AD pathology (note: could SVD be CAA?)
	337 NCI			
	547 AD			
	30 VCI			The presence of lacunes was associated with higher Aβ42 in vascular dementia (standardized beta = 0.17, *P* = 0.07) and lower tau in AD (standardized beta = -0.07, *P* = 0.05) but there were no effects for Aβ42 or phosphorylated tau181 in AD (note: could SVD with lacunes be arteriolosclerosis?)

Aβ, amyloid-beta; AD, Alzheimer disease; ADNI, Alzheimer’s Disease Neuroimaging Initiative; CAA, cerebral amyloid angiopathy; CI, cognitively impaired; CSF, cerebrospinal fluid; FDG, [^18^ F]fluorodeoxyglucose; MB, microbleed; NCI, no cognitive impairment; NINDS-ADRDA, National Institute of Neurological Disorders and Stroke-Association Internationale pour la Recherche et l’Enseignement en Neurosciences; NINDS-AIREN, National Institute of Neurological Disorders and Stroke-Association Internationale pour la Recherche et l’Enseignement en Neurosciences; SVD, subcortical vascular dementia; VBI, vascular brain injury; VCI, vascular cognitive impairment; VRF, vascular risk factor; WMH, white matter hyperintensity.

In the Alzheimer Disease Neuroimaging Initiative, VRFs were not associated with AD biomarkers (that is, CSF amyloid, [^18^ F]fluorodeoxyglucose (FDG) PET uptake or MRI hippocampal atrophy). In normal controls, progressive increases in WMH over time were associated with greater decline in executive function and lower FDG PET uptake (Table 4) [50]. One should keep in mind that the Alzheimer Disease Neuroimaging Initiative is focused on AD not CVD. The Framingham Coronary Risk Profile scores for the cohort were relatively low, approximately 18% across normal cognition, mild cognitive impairment and AD groups. Nonetheless, no interactions were noted between VRFs and AD biomarkers.

## Other possible pathophysiological interactions between VRFs, CVD, and AD pathology

Converging evidence from epidemiologic [51-53], neuropathologic, amyloid PET, and CSF biomarker studies show that VBI and AD exert additive adverse effects on cognitive health (Figure 1). Do VRFs and CVD merely increase the co-occurrence of two separate processes (that is, AD and silent/symptomatic VBI), which shifts the syndromal diagnosis of dementia and AD earlier (reviewed in [9,54])? Or do VRFs and CVD potentiate AD-specific pathophysiological pathways, such as amyloidopathy and tauopathy? Several mechanisms have been postulated by which Aβ may be cleared in the brain: 1) enzymatic degradation (for example, neprilysin, insulin degrading enzyme) by microglia and astrocytes; 2) active transport from brain to blood through an endothelial lipoprotein receptor-related protein-mediated process [55-57]; and 3) passive transport through a perivascular ‘lymphatic-like’ drainage system of the brain [58,59]. Recently, associations between the accumulation of Aβ on PiB PET scans with serum cholesterol and aortic arterial stiffness have been described [46,47,60]. However, direct associations between Aβ clearance and levels of brain cholesterol or cerebral arteriolar/venous stiffness in human beings have yet to be shown. Thus, interactions at the pathophysiological level between VRFs/CVD and AD pathology, while plausible, are still unresolved.

## Type of cerebrovascular disease matters for prevention and treatment

VCI and VBI refer to phenotypes rather than treatable etiologies. It should be apparent that clinical diagnosis must go further to identify the underlying type of CVD. While there are many possible types of CVD, the three major types are atherosclerosis, arteriolosclerosis, and CAA. Risk factors for atherosclerosis and arteriolosclerosis overlap considerably and include hypertension, diabetes mellitus, and hyperlipidemia, which are easily measured and effectively treated with medications and lifestyle modifications.

The location of some, but not all, structural imaging features may help to distinguish underlying arteriolosclerosis from CAA. WMHs are associated with both hypertensive arteriosclerosis and severe CAA, with evidence of a more posterior location favoring CAA [61,62]. Lacunar infarcts are more likely to be associated with arteriolosclerosis than with CAA [21]. The regional distribution of MBs may help to differentiate SVD and CAA; the occurrence of MBs in deep gray nuclei suggests SVD, while MBs in lobar or cortical regions suggest CAA [63,64]. Though promising, the sensitivity and specificity of categorizing strictly lobar as CAA, strictly deep as hypertensive vasculopathy, or both lobar and deep as coexisting CAA and hypertensive vasculopathy has not yet been studied [64].

Evidence-based treatment recommendations for VCI have been recently reviewed [1]. Also, based on comprehensive reviews of the literature, and assuming a causal relationship and intervention at an appropriate age to reduce the prevalence of each of seven risk factors by 10% per decade, it has been estimated that the prevalence of AD/dementia could be reduced by 8.3% worldwide by 2050 [53]. This would translate to 1.1 to 3 million AD/dementia cases worldwide and 184,000 to 492,000 cases in the USA [65]. These risk factors include five vascular risk factors (that is, midlife hypertension, diabetes mellitus, mid-life hyperlipidemia, smoking, sedentary life style), as well as depression and low educational attainment. Arguably the epidemiological diagnosis of AD may well include cases with subclinical VBI or mixed AD/VBI. Regardless of etiological label, assuming internal consistency in the definitions of risk factors and the diagnosis of dementia subtype, the epidemiological data present a valid and worthy public health objective, namely a 10% reduction in VRFs for 10 years for an 8% reduction in dementia.

The major risk factor for CAA is the ApoE ε4 allele, which can be determined readily by genotyping, though is not yet recommended by best practice guidelines. Strategies to reduce risks related to the ApoE ε4 genotype have been explored in cultured neurons [66] and in animal models of AD [67]. Bexaratone reduces amyloid plaques in animals, but no clinical trials have been conducted in humans.

## Conclusion

The co-occurrence of AD and VBI in elderly persons is very common. There is now a large body of evidence showing that AD and VBI lead in an additive and independent fashion to cognitive dysfunction. In AD, there is a characteristic pattern of tau-related, neurofibrillary neurodegeneration spreading from the medial temporal lobe to other multi-modal association areas and a corresponding pattern of memory loss spreading to other cognitive domains. By contrast, there is tremendous variation in the neuropsychological profile associated with VBI. In the SVD subtype of VCI, executive dysfunction often equals or may exceed memory impairment, but depending on location all varieties of cognitive impairment may ensue.

WMHs, small and large infarcts and hemorrhages are visible on structural MRI and CT imaging and currently serve as the most reliable marker for VBI. However, microinfarcts, which have been most strongly related to cognitive impairment in neuropathology studies, continue to elude clinical detection. The validation of amyloid PET imaging adds considerable specificity for the diagnosis of AD, beyond the long recognized atrophy of the hippocampus. The addition of tau PET imaging is expected shortly. Global measures of atrophy on MRI are important markers of overall brain injury, but cannot be used reliably to determine etiology.

Longitudinal studies with repeat neuropsychological testing support multivariate approaches to model the effects of various types of pathology on dementia risk and cognitive decline. Once comparable measures of AD and VBI pathology are available from *in vivo* neuroimaging studies, we can anticipate that one day dichotomous classifications will be replaced by more sophisticated modeling. Still, the best models available today predict less than half of the variance in cognitive performance.

For prevention and treatment, it is important to bear in mind the type of CVD underlying VBI and VCI, as well as to consider that subclinical CVD and VBI may contribute additively to cognitive impairment in patients with AD. Epidemiological data suggest that attention to ‘life’s simple seven’, referring to seven health factors and lifestyle behaviors identified by the American Heart Association that include being physically active, eating foods low in cholesterol and saturated fats, monitoring high blood pressure and blood sugar, maintaining a healthy weight, controlling cholesterol, and avoiding tobacco smoking, can significantly reduce the risk of dementia.

## Note

This article is part of a series on *Cerebral multi-morbidity of the aging brain* edited by Johannes Attems and Julie Schneider. Other articles in the series can be found at http://alzres.com/series/cerebral_multimorbidity.

## Abbreviations

Aβ: Amyloid-beta
AD: Alzheimer disease
ApoE: Apolipoprotein E
BLSA: Baltimore Longitudinal Aging Study
CAA: Cerebral amyloid angiopathy
CFAS: Cognitive Function and Ageing Study
CSF: Cerebrospinal fluid
CT: Computerized tomography
CVD: Cerebrovascular disease
FDG: [^18^ F]fluorodeoxyglucose
HAAS: Honolulu Asia Aging Study
MAP: Memory and Aging Project
MB: Microbleed
MBI: Microscopic brain infarct
MRI: Magnetic resonance imaging
PET: Positron emission tomography
PiB: Pittsburgh Imaging Compound B
ROS: Religious Orders Study
SE: Standard error
SVD: subcortical vascular dementia
VBI: Vascular brain injury
VCI: Vascular cognitive impairment
VRF: Vascular risk factor
WMH: White matter hyperintensity
